# Focus on geriatric proximal femur fractures: factors that influence the outcome

**DOI:** 10.1007/s00068-022-01954-7

**Published:** 2022-04-11

**Authors:** Philipp Kobbe, Miguel Pishnamaz, Frank Hildebrand

**Affiliations:** Department of Orthopaedics, Trauma and Reconstructive Surgery, University Hospital RWTH Aachen, Pauwelsstrasse 30, 52074 Aachen, Germany

Due to demographic trends, the importance of optimal treatment of proximal femur fractures in elderly patients will undoubtedly continue to increase. The clinical outcome for this vulnerable patient group depends on many factors, some of which we as surgeons may influence (e.g., timing of surgery and intraoperative blood loss). However, patient-specific factors (e.g., age, nutrition status, and cognitive disorders) are also of upmost importance and may raise our suspicion for a complicative course.

In this issue, several authors highlight different surgery-related and patient-specific factors that may influence the perioperative course of geriatric patients with proximal femur fractures.

Kristen et al. [1] retrospectively reviewed 634 patients with hip fractures and showed that surgical treatment after 48 h was the only factor that significantly increased the length of hospital stay. In cases where patients were treated within 48 h, the length of hospital stay was mainly influenced by a higher ASA score, anticoagulant therapy and the type of surgery. Based on their results, the authors emphasized the importance of treatment-related and organizational factors to minimize the delay until surgery, thereby beneficially influencing the clinical course of the patient.

In the context of surgical timing, Forssten et al. [2] investigated the impact of out-of-hours surgery on mortality in patients undergoing hip fracture surgery. The authors were able to show that out-of-hours surgery was associated with an increase in both 30- and 90-day mortality among hip fracture patients if treated with arthroplasty but not in those patients treated with internal fixation. As potential reasons, limited out-of-hours availability of resources to control vital signs and the occurrence of other complications was discussed, which might be more relevant for arthroplasty, which clearly represents the more invasive surgical intervention. Therefore, the authors conclusion was to avoid procedures involving arthroplasty in the out-of-hours period.

In addition to the above reported impact of timing, Weingärtner et al. [3] highlighted a further risk in the context of arthroplasty: bone cement implantation syndrome (BCIS). In their study, they investigated the rate and clinical impact of BCIS in patients with proximal femur fractures treated with a cemented hemiarthroplasty. Thirty-seven percent of patients showed symptoms of BCIS, which was associated with a significantly increased risk for cardiovascular complications and in-hospital mortality. The identified risk factors for BCIS were age, the absence of a femoral borehole and ASA status.

In their randomized double-blinded trial, Zhang et al. [4] evaluated the efficiency of intravenous tranexamic acid (IV-TXA) on hidden blood loss caused by postoperative hyperfibrinolysis in patients treated for intertrochanteric fractures. Their data indicated that IV-TXA 10 min before and 3 h after incision effectively reduced hidden blood loss and the need for erythrocyte transfusion without a significant increase in thromboembolic events. As patients with intertrochanteric fractures are usually frail and suffer from previous anemia, it might be assumed that the proposed therapy has the potential to reduce an anemia-related delay of functional recovery.

A retrospective, observational study evaluated whether beta-blockers therapy provided a survival benefit following hip fracture surgery [5]. Ahl et al. included 134,915 patients from the Swedish Hip Fracture Register, with almost 40% of patients having ongoing beta-blocker therapy. Although patients in this group were significantly older, they showed a significant survival benefit with a 72% relative risk reduction in 30-day all-cause mortality. The authors concluded that their findings clearly encourage further studies with an interventional design to validate the presented relationship.

Finally, Becker et al. [6] reviewed several patient-specific risk factors for outcome after proximal femur fractures. They especially focused on the general medical condition of the patient upon admission, which may indicate which patients are at increased risk for adverse outcomes.

We hope that this issue will provide you with useful information regarding your daily practice with this vulnerable patient group.
